# Chronic Salmonella Osteomyelitis in a Diabetic Patient

**DOI:** 10.7759/cureus.1285

**Published:** 2017-05-29

**Authors:** Stella Pak, Cindy Pham

**Affiliations:** 1 Internal Medicine, University of Toledo Medical Center

**Keywords:** salmonella enteritidis, chronic osteomyelitis, surgical excision

## Abstract

Salmonella osteomyelitis in patients without hemoglobulinopathy is quite uncommon. Osteal involvement is seen in only 0.8% of all Salmonella infection cases. We describe the case of a 67-year-old diabetic woman who developed Salmonella osteomyelitis and subsequently underwent a surgical excision of a tibial lesion followed by two months of intravenous (IV) antibiotic therapy. The patient responded very well to the treatment. This case is exemplary for the successful treatment of chronic Salmonella osteomyelitis in a diabetic patient with a vascular complication.

## Introduction

*Salmonella enteritidis* uses eggs and poultry as vehicles to infect its hosts. In immunocompetent individuals, the manifestation of the food-borne infection is mostly limited to self-limited gastroenteritis. *S. enteritidis* is a very rare bacterial isolate in osteomyelitis [1]. Therefore, a recent history of gastroenteritis can be a diagnostic clue for acute Salmonella osteomyelitis. Hemoglobinopathies such as sickle cell disease (SCD) and thalassemia are strong risk factors for osteomyelitis [2]. Immunosuppressed status including diabetes mellitus, human immunodeficiency virus (HIV), chronic steroid use, and peripheral vascular disease (PVD) also increase the risk of bone infection. In addition, approximately 50% of diabetic patients suffer peripheral neuropathy, predisposing the patients to the development of unrecognized soft tissue infections, which can contiguously spread to the bone [3]. We herein present the case of a 67-year-old diabetic woman who developed Salmonella osteomyelitis and subsequently underwent a surgical excision of a tibial lesion followed by two months of intravenous (IV) antibiotic therapy.

## Case presentation

A 67-year-old woman presented with worsening intermittent right leg pain for the past year. The patient reported extreme right leg pain associated with a thigh swelling, which for the past two months has been draining fluid. Her past medical history was significant for insulin-dependent diabetes mellitus (IDDM) type II and PVD.

Physical examination revealed a fluctuant mass (2 cm in width and 2 cm in length) located on the anterior superior third of the tibia with purulent drainage. She had a full range of motion of both knees and ankles. The dorsal pedis and posterior tibialis pulse were palpable and symmetrical.

**Figure 1:**
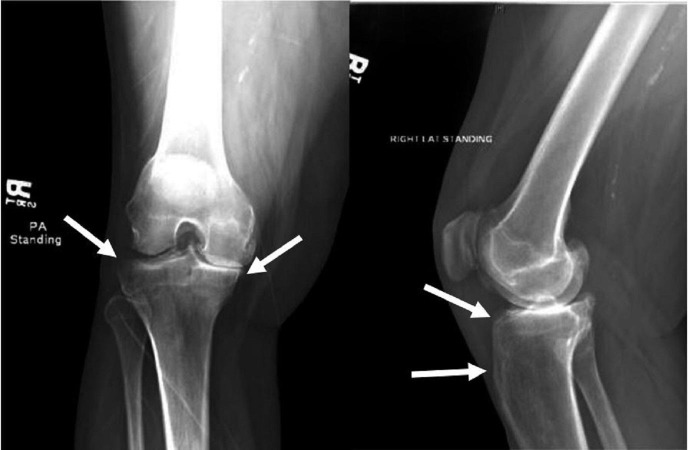
Roentgenogram of right knee. Note the bone expansion and marked sclerosis.

Both the bone biopsy and the pus swab from the drained material grew *Salmonella enteritidis*; however, her blood and urine culture were negative for the bacterium. X-ray of the right tibia showed a 4-cm cortical defect with several densities within the medullary canal. The defect was in the upper one-third of the tibial diaphysis just inferior to the metaphysis (Figure 1).

Our patient was immediately started on IV levofloxacin (500 mg daily) therapy. Due to the chronicity of the infection, a partial surgical excision was performed on the proximal tibial lesion. The bone biopsy from the operation demonstrated marrow spaces that were partially replaced by fibrous tissues and mononuclear cells; this was compatible with chronic osteomyelitis. Postoperatively, she achieved uneventful wound healing and clinical recovery. Subsequently, she was discharged on the sixth day of hospitalization.

She was maintained on levofloxacin therapy for two months and received physical and occupational therapy on an outpatient basis. At one-year follow-up, the patient was in good condition without recurrence of osteomyelitis.

## Discussion

The common pathogens in osteomyelitis are *Staphylococcus aureus* and *the Streptococcus* species. It is crucial to consider the possibility of Salmonella osteomyelitis in patients with known risk factors including hemoglobulinopathy, diabetic mellitus, and PVD. Our patient had two of those three risk factors – 30 years of diabetes that had been complicated by PVD. Identification of the pathogen involved is critical in the management of osteomyelitis because the recommended therapy varies based on the causative organism and its susceptibility. For methicillin-sensitive *Staphylococcus*, nafcillin, oxacillin, or cefazolin is the first line of therapy. Vancomycin or linezolid is preferred for methicillin-resistant *Staphylococcus*. For the *Streptococcus* species, penicillin is recommended. The preferred therapy for Salmonella is fluoquinolones, such as ciprofloxacin or levofloxacin [3]. In accordance with current guidelines, our patient was treated with levofloxacin for two months.

Surgical excision of the affected area is recommended for chronic osteomyelitis with necrotic tissues or cases involving surgical hardware or refractory to antibiotic therapy [3]. For our patient, surgical excision was performed due to the chronicity of bone infection. Our patient responded extremely well to the surgical excision and antibiotic therapy. The successful therapy may have helped prevent a recurrence of osteomyelitis.

## Conclusions

We report a clinical case exemplary for the successful treatment of chronic Salmonella osteomyelitis in a diabetic patient with a vascular complication. For patients with known risk factors, it is important for clinicians to maintain a high index of suspicion for Salmonella osteomyelitis. As the prevalence of diabetes continues to increase, clinicians should expect to see more cases of osteomyelitis. Therefore, we need to familiarize ourselves with known risk factors for rare pathogens of osteomyelitis.

## References

[1] Oki M, Ueda A, Tsuda A, Yanagi H, Ozawa H, Takagi A (2016). Salmonella enterica serotype enteritidis vertebral osteomyelitis and epidural abscess complicated with meningitis. Tokai J Exp Clin Med.

[2] McAnearney S, McCall D (2015). Salmonella osteomyelitis. Ulster Med J.

[3] Hatzenbuehler J, Pulling TJ (2011). Diagnosis and management of osteomyelitis. Am Fam Physician.

